# Exploring the experiences of people with obesity and post-bariatric surgery patients after three months using the mindful eating reflective practice: An interpretative phenomenological analysis

**DOI:** 10.1177/02601060241281779

**Published:** 2024-09-23

**Authors:** Michael Mantzios, Henna Bahia, Misba Hussain, Rebecca Keyte, Helen Egan, Rachel Strachan, Abd A Tahrani

**Affiliations:** 1 College of Psychology, 1725 Birmingham City University, UK; 2 University Hospitals Birmingham, UK; 3 Institute of Metabolism and Systems Research, 1724 University of Birmingham, Birmingham, UK; 4 Department of Endocrinology and Diabetes, 1732 University Hospitals Birmingham NHS Foundation Trust, Birmingham, UK; 5 Centre for Endocrinology, Diabetes and Metabolism, Birmingham Health Partners, Birmingham, UK

**Keywords:** Obesity, bariatric surgery, mindful eating interventions, self-Compassion, interpretative phenomenological analysis

## Abstract

**Background:** Experiential dimensions of Mindful Eating Practices are scarce in the literature. Aim: The study focuses on thirteen individuals with clinical obesity and nine post-bariatric surgery patients who engaged in MERP over three months. **Methods:** The present research utilized Interpretative Phenomenological Analysis (IPA) as the analytical framework of interviews. **Results:** Four overarching themes emerged from the analysis: 1. “Enhanced Awareness of Eating": This theme underscores MERP's central emphasis on cultivating heightened mindfulness during food consumption, highlighting the importance of being present at the moment while eating; 2. “Facilitating the Transition to Healthier Eating Habits": This theme explores how MERP influences participants’ dietary choices, eating pace, portion control, and overall enjoyment of meals. It reveals that MERP encourages individuals to reflect on their eating habits and transition towards healthier choices; 3. “Diverse Perspectives on Satisfaction with MERP": Within the context of MERP, participants held varied interpretations of satisfaction. Some encountered practical limitations or engaged in reflective self-examination, while others found sensory satisfaction, enhancing their overall eating experiences; and 4. “Utilization and Development of MERP": This theme delves into participants’ patterns of using MERP. It reveals a tendency to avoid MERP in the morning, a gradual decline in its usage over time, and a preference for an electronic version of the practice. **Conclusion:** The MERP shows promise in improving overall eating habits by enhancing enjoyment of food, increasing awareness of body cues, promoting healthier choices, and encouraging mindful eating practices. These findings provide valuable insights for future research and the refinement of clinical tools aimed at effective weight management and the promotion of sustainable healthy eating practices by effectively addressing a significant gap in our understanding of the experiential facets of eating practices.

## Introduction

The World Health Organization emphasizes the need for obesity prevention due to its significant impact on individuals, society, and the healthcare system (WHO, 2022). Obesity is a global health concern affecting metabolic, physical, and mental health. In England, approximately 29% of adults are classified as having obesity (NHS, 2021). While behavioural lifestyle interventions demonstrate efficacy in achieving modest weight loss (3–10%), the challenge of weight regain persists for most patients (Yanovski and Yanovski, 2014).

Bariatric surgery emerges as a highly effective intervention, recommended in the UK for individuals with severe obesity (BMI ≥ 40 kg/m² or BMI 35–39 kg/m² with obesity-related complications) (NICE, 2014). This surgery not only positively influences obesity-related complications, but also addresses conditions like type 2 diabetes, obstructive sleep apnea, hypertension, cancer, and cardiovascular disease (Christou et al., 2004; Mechanick et al., 2013; Singh et al., 2020). Research shows that bariatric surgery can enhance both physical and psychological well-being, leading to improved quality of life and variable mental health outcomes (e.g., improvement in depression symptoms dependent on weight outcomes; Smith et al., 2020) post-surgery (Castaneda et al., 2019; De Zwaan et al., 2002; Karlsson et al., 2007; Kolotkin et al., 2012). Despite the proven benefits of bariatric surgery, approximately 21% of patients experience weight regain after the initial post-surgery weight loss (King et al., 2020; Voorwinde et al., 2020). The determinants of weight regain post-surgery are not fully understood (Heinberg et al., 2020), and the literature suggests that the psychological benefits of bariatric surgery may have a limited duration, with effects diminishing around two years post-surgery, returning to pre-surgery levels (Buddeberg-Fischer et al., 2006). While a solution is readily available, the implications of providing additional behavioural support for both obesity and in the post-bariatric surgery phase are significant for the self-regulation and sustainability of weight.

One solution that has been proposed is mindful eating (Hussain et al., 2022). The history of mindful eating and mindful eating interventions stretches over two decades. Mindful eating is derived from secular mindfulness literature and interventions, with the intention of assisting eating behaviours (Mantzios, 2021). Hanh and Cheung (2011) advocated for the broad definition of mindful eating, encompassing the incorporation of mindfulness practices into eating experiences (Allirot et al., 2018; Mantzios et al., 2018a, 2018b). Much of the research have focused on cross-sectional and trial data (Dalen et al., 2010; Mantzios and Wilson, 2014; Miller et al., 2014; Hussain et al., 2022), with limited data on user experience, attitudes and beliefs, which may be instrumental in understanding the sustainability of interventions. Furthermore, there are variations of definitions, interventions and practices, which at times are inclusive of other factors such as self-compassion (Mantzios and Wilson, 2015a, 2015b), and at other times exclusive of fundamental aspects of mindfulness and mindful eating such as non-judgemental awareness (Tapper and Seguis, 2020; Keyte et al., 2020). This introduces uncertainty on how some practices work for the users, and impacts the ability to replicate findings across different mindful eating paradigms. In this research, we aimed to explore the Mindful Eating Reflective Practice (MERP), which has been an outcome of several years of research and experimentation (Hussein et al., 2017; Mantzios, 2023; Mantzios and Wilson, 2015a, 2015b).

The journey started with research that explored the utilization of the Mindful Construal Diary (MCD), developed by Mantzios and Wilson (2014), as an alternative to traditional mindfulness meditation for weight regulation and enhanced well-being. The MCD combines mindfulness, self-compassion, and construal level theory (CLT) principles, emphasizing the importance of concrete construals in fostering present-moment attention and non-judgment. Concrete construals, as opposed to abstract ones, encourage attention to the “how to” eat, rather than the “why”, thus promoting mindfulness and self-compassion (Hussein et al., 2017). Importantly, self-compassion, characterized by non-judgment, non-evaluation, and acceptance, plays a pivotal role in this process by adding an additional layer of emotion regulation (Sirois, 2015), and enabling the resilience to the struggles of the present moment experienced through mindfulness (Evans et al., 2018; Hollis-Walker and Colosimo, 2011). Mantzios and Wilson (2014) incorporated self-compassion into the MCD, transforming it into an event-based diary that simplified the practice, making it more accessible than meditation. Research has demonstrated the effectiveness of the MCD in weight maintenance compared to mindful self-compassion programs delivered via meditation (Mantzios and Wilson, 2015a, 2015b).

Subsequent studies introduced variations of the MCD, such as the MERP, which encouraged mindful eating without the diary format (Hussain et al., 2021a, 2021b). These adaptations produced positive outcomes, including increased mindfulness, self-compassion, and decreased anxiety, offering insights into the mechanisms behind weight regulation. Additionally, experiments using the raisin exercise and the MERP found that both techniques reduced chocolate consumption compared to control conditions, highlighting their potential for improving eating behaviours with highly palatable foods (Mantzios et al., 2020a, 2020b). A recent study by Hussain et al. (2021c) enhanced the MERP by introducing a self-distanced perspective, ultimately assisting individuals in reducing emotional eating and consuming less chocolate (Hussain et al., 2021a, 2021b). In summary, extensive research into the MCD and its variations strengthens the evidence for their potential as effective promotions of mindful eating, self-compassion, and better weight regulation, with the added benefit of being more accessible than traditional meditation practices. Mantzios et al. (2020a, 2020b) reiterated that priming or nudging individuals to eat more mindfully (Mantzios, 2023) could offer a more practical and sustainable approach to integrating mindful eating into everyday life (Mantzios and Giannou, 2018). This reflective approach focuses on ease of use, long-term adherence, and sustainability, potentially facilitating the development of healthier eating behaviours over time.

To date, the MERP has only been explored through quantitative research methods (Hussain et al., 2021a, 2021b, 2021c; Mantzios, 2021; Mantzios et al., 2020b), which restrict the extent to which participants can comment on their experiences, attitudes and beliefs and, prevent any further development of the MERP. Qualitative methods, on the other hand, allow for an in-depth understanding of experiences, opinions, thoughts and perspectives within a social context (Barker et al., 2015; Rahman, 2020).

Therefore, a qualitative approach was deemed appropriate for the present study, as this would enable a detailed investigation of the phenomena (e.g., user experience, thoughts, attitudes, and beliefs) which are not easily quantifiable (Rahman, 2020). Additionally, a qualitative approach allows for the emergence of unanticipated findings (Barker et al., 2015), which in the context of user experience could be valuable. Although several facets of the user experience could have been explored, two specific elements were focused on; these were the participants’ thoughts, attitudes and perceptions of the eating experience, and the practical issues experienced when using the MERP.

## Methodology

### Participants

Adults ages 18 years or above, cared for by the Specialist Weight Management Service and/or the Specialized Bariatric Surgical Service operated at the Heartlands Hospital, University Hospitals Birmingham (UHB) NHS Trust in Birmingham, UK were eligible to participate. Participants who met the eligibility criteria were recruited during clinic attendance. Following their clinical appointments, patients were approached by the second author of this manuscript for participation. The recruitment process spanned 10 months and continued until data saturation was achieved.

Overall, twenty-two participants were recruited. Thirteen female participants (age range: 30–64 years, mean: 44 years) from the weekly obesity group treatment sessions were recruited with a mean BMI of 44.36 (*SD ± *6.66), while no male participants were willing to participate. From the post-bariatric services, nine participants (females: 6, males: 3, age range: 31–67 years, mean: 54 years) were recruited with a mean BMI of 40.91 (*SD ± *12.11). The specialized bariatric surgical services provide weight management, ranging from pre-operative assessment to post-operative care. Participants actively participated in the program by attending appointments with a specialized bariatric dietician every three months. Recruitment for the research study was voluntary and happened during these appointments. We offered participation in the research as an additional support element to those who were interested. The dietician's role encompassed monitoring weight and eating behaviour, addressing any surgical complications, and delivering crucial nutritional education, which is standard care for all patients.

A summary of participant demographic characteristics is provided in Table 1.

**Table 1.** Participant demographic characteristics.

Sample	Pseudonym	Age (years)	BMI (kg/m^2^)	Employment status
Patients with Clinical Obesity (CO) (*n *= 13)	Sue	30	40.4	Employed
	Becky	61	51.3	Carer
	Angie	53	48.3	Employed
	Claire	38	49.5	Employed
	Danielle	33	42.3	Employed
	Lynn	47	45.0	Unemployed
	Debbie	48	53.9	Unemployed
	Jenny	52	35.4	Employed
	Mandy	50	34.9	Employed
	Emma	64	46.2	Retired
	Mary	45	48.9	Disabled
	Charlotte	43	47.5	On sick leave
	Kerry	60	33.1	Employed
Patients with Post-Bariatric Surgery (PBS) (*n *= 9)	Kathy	50	36.8	Employed
	Maggie	55	30.0	Retired
	Asha	55	31.2	Employed
	Tom	60	30.1	Carer
	Lucy	56	40.4	Retired
	Katie	63	48.5	Retired
	Andy	54	68.2	Retired
	Hannah	31	44.9	Employed
	Dean	67	38.1	Retired

### Mindful eating reflective practice (MERP)

In this study, MERP was used for three months among all participants before the interview sessions. Eating-specific mindful exercises, like the Mindful Construal Diary (MCD) developed by Mantzios and Wilson (2014), appear to be more effective for promoting healthier eating behaviours and weight loss than generic mindfulness practices. The MCD combines concepts from both mindfulness and construal-level theory. Research has shown that using the MCD, such as answering mindful construal questions while eating, can assist with weight regulation (Mantzios and Wilson, 2014). Additionally, reflecting on the mindful construal method as seen in the MEBP has been found to improve mindfulness, self-compassion, and reduce anxiety levels (Hussein et al., 2017; Hussain et al., 2021a, 2021b, 2021c; Mantzios, 2023; Mantzios et al., 2020a, 202b), providing continuity and advancement of the MCD. The latest research showed the development of the MERP and the alignment of theories and definitions to practices that focus on Mindful Eating Behaviour (Mantzios, 2021; Mantzios, 2023). Mantzios’ (2023) recent research introduced the latest practice, which is the continuation of the MCD/MERP, which can be found in the supplementary materials. Across all the practices, historical and current, the general instructions aimed to equip participants with the skills for mindful eating remained constant. These included proper implementation of the technique by (a) heightened awareness of the senses during the practice, and, (b) strategies for bringing attention back to the present moment when the mind wanders.

### Semi-structured interview

The semi-structured interviews started by investigating participants’ experiences, opinions, thoughts, attitudes and beliefs of the MERP which they had been requested to engage with for three months prior to participating in the interview. The interview schedule then became more specific where participants were asked to describe their usage of the MERP on a daily, weekly and monthly basis, and well as provide insight into which meals participants tended to use the MERP most, thus illustrating barriers to using the MERP at other meals. The qualitative methodology allowed for the exploration of the defined areas, and elaboration by participants on other related topics, with question wording and order being contextual and in response to the participants’ developing accounts (Braun and Clarke, 2013; Colley, 2014).

The semi-structured interview schedule was carried out according to the existing literature and discussions with healthcare professionals working within the UHB weight management and bariatric departments. All interviews were conducted in person in hospital clinic rooms, with the interviews lasting an average of 40 min.

#### Ethical approval

Ethical approval was obtained by the National Research Ethics Service (NRES) (Approval references: 17/WS/0065; 17/LO/0653). Written informed consent was obtained from all individual participants included in the study, with all participants provided with a pseudonym by the researcher.

## Data analysis

The recordings of the interviews were transcribed verbatim. The data was analysed using Interpretative Phenomenological Analysis (IPA) following the procedure outlined by Smith et al. (2022). IPA emphasises a phenomenological approach that enables researchers to explore participants’ unique accounts and perceptions of a phenomenon in its own terms (Smith et al., 2022), providing a detailed examination of shared experiences among participants (Alase, 2017). IPA upholds the importance of individual experience to access underlying perceptions, position or context about phenomena (Heotis, 2020) and is therefore suitable for exploring the experience of using the MERP.

Single case analysis was initially conducted by one researcher, and subsequently revised and evaluated by a collaborator until they reached an agreement for each code. The same procedure was repeated for each transcript. Emergent themes were noted for all transcripts before being clustered with similar emergent themes from other transcripts to create superordinate themes. Similarly, the researchers evaluated whether the themes accurately represented the data, whether there was enough data to support each theme, theme distinctiveness, and whether each theme was relevant to the study aims. The collaborations helped to ensure the reliability of the data generated resulting in a final list of master and superordinate themes (Table 2).

**Table 2.** Master and superordinate themes.

Master themes	Superordinate themes
Greater awareness leads to self-regulation and self-monitoring	Awareness induced guilt
	An increased awareness of internal sensations
	An increased awareness of food
Transitioning to a healthier and more adaptive way of eating	Healthier consumption
	Slowing down consumption
	Assisting with portion control
Eating satisfaction: A varied experience	Enhanced enjoyment and rediscovery of eating pleasure.
	A decline in enjoyment of the eating experience
MERP usage and development	An unsocial eating experience
	Morning and Evening usage
	Nudging and habitual use
	Electronic format

To further ensure the validity of the data, the lead researcher kept a reflective diary through data collection and analysis, with data triangulation being achieved by the research findings and the responsible supervisory team.

## Results

Four master themes (Table 2) were constructed from the data set providing an interpretation of the participants’ experience of using the MERP. The themes discussed do not cover all elements of participants’ experience of using the MERP but were chosen due to their relevance to the research purpose.

### Master theme: greater awareness leads to self-regulation and self-monitoring

It is clear within this initial theme that participants developed a heightened awareness of their internal cues particularly regarding hunger and fullness. Participants such as Mary, Kerry, and Jenny described how engagement with the MERP increased their ability to discern physiological hunger from other impulses, emphasizing the role of mindful awareness in guiding their eating decisions.

*Mary [45 years] (CO): “when I have the thoughts that I’m actually full (.) I would stop whereas normally I would carry on – (104.23)”*

*Kerry [60 years] (CO): “if I felt hungry I would think am I really hungry or (.) do I just want a drink - (48.10)”*

In addition to being more aware of genuine hunger, several participants such as Maggie, Kathy and Tom noted increased sensory attention to food, becoming more appreciative of the taste, texture, and smell of their meals, transforming the act of eating into an enjoyable experience.

*“Kathy [50 years] (PBS): It made me more mindful of what I was cooking and that I actually do prefer to have fresh vegetables and everything else because I like the taste and I like the texture – (38.11)”*

*Maggie [55 years] (PBS): “I never used to really like veg but now I crave veg – (50.15” […] All I keep in my head is the smell texture taste and that really works for me – (54.16”*

*Katie [63 years(PBS)]: You sort of start thinking about the taste, the appearance, the portion size – (72.24)”*

Overall, the heightened awareness of the eating context directed participants’ attention to their food choices, portion sizes, and internal cues, facilitating self-regulation and healthier eating behaviours.

### Master theme: transitioning to a healthier and more adaptive way of eating

This theme provides insight into participants’ experiences with the MERP, highlighting the positive impact of using the MERP on various aspects of participants’ eating habits and overall wellbeing. This included healthier consumption, slowing down consumption, and assisting with portion control.

Many participants, including Lucy, Andy, Kerry, and Mandy, discussed how using the MERP has led them to make healthier food choices, shifting away from less nutritious options towards fresh, wholesome foods, reflecting an adaptive change in their dietary habits.

*Lucy [56 years] (PBS): “Since I’ve been doing that [using the MERP] I can honestly say that I have not had any fried food – (119.3)”*

*Mandy [50 years] (CO): “You try to look for the healthier options or try to actually instead of buying pre prepared meals you’d try to prepare your own meals from fresh - (116.24)”*

This transition was not just about consuming more healthful foods like fresh vegetables, but also entailed changes in their overall eating habits. Mandy uses ‘try’ several times which denotes the effort required to change habits and how this comprises several preparation stages. Participants became more mindful of their food choices, leading them to opt for healthier options as seen in the quotes from Lucy and Mandy. The MERP helped those recognize enjoyable foods and nourished their bodies.

In addition to being more mindful of food choices, participants, such as Hannah and Kathy, have noted that the MERP has encouraged them to eat at a more measured pace, fostering a sense of mindfulness during meals.

*Hannah [31 years(PBS)]: “Because you’re having to like stop and think and eat at the same time you just kinda like slow down – (22.7)”*

*Hannah [31 years] (PBS): “beforehand I would rush and tend to gobble everything down but now I’ve actually slowed down– (120.50)”*

In the methods section, the study describes how the MERP involved participants reading and reflecting on questions. These questions primed them to focus on their sensory experiences while eating naturally; a focus which encouraged participants to slow down while eating. This is particularly a positive change for post-operative bariatric patients as eating slowly is recommended to aid digestion, and has implications for responding to internal cues and satiety.

As well as “slowing down”, participants such as Asha, Maggie, Jenny, and Emma explained how the MERP has prompted them to consider portion sizes more carefully, thereby aiding in portion control and potentially preventing overeating.

*Emma [64 years(CO)]: “I think it [the MERP] does make you more conscious of the amounts of foods you are eating (.) and I think in that respect I did (.) cut down on my portion size - (46.12)”*

*Maggie [55 years(PBS)]: “That [the MERP] really helped to keep me on track you know even if I wanted to try and say oh yeah I’ll have a little bit more than that and override my stomach, but if I eat too much I get really bad pains – (30.9)”*

Maggie describes a conflict between body and mind, ‘overriding’ the body's needs in order to accommodate the desire to eat more despite painful consequences. This highlights how the MERP was used by participants as a means of portion control, focusing on the amount of food that the body needs.

### Master theme: eating satisfaction: a varied experience

This theme highlights how individual the experience of using the MERP is, with participants providing contrasting accounts of their eating experience. Some participants found eating more enjoyable following the MERP, primarily due to increased awareness of food and the act of eating. Tom, Dean and Kathy were grateful for their heightened sensory awareness, describing a newfound appreciation for the diverse tastes and textures of their food, highlighting how mindful eating has transformed their meals into more satisfying experiences.

*Mary [45 years] (CO): “I think it [the MERP] puts that enjoyment there (.) back into the food - (120.28)”*

*Kathy [50 years] (PBS): “Food now is not just fuel it's something that I enjoy – (147.31)”*

*“Lucy [56 years] (PBS): “I’d have salad because I have to # now I have salad because I enjoy it – (62.14)”*

*Dean [67 years] (PBS): “It's far more enjoyable because you are getting different tastes different textures, I didn’t care about texture before but I do now – (118.25”*

The ways in which enjoyment in food was described as increased, gives insight into prior experiences where food was not enjoyable, it was seen as something that was necessary to live, and healthy foods, such as salad, were not eaten out of choice.

Other participants, such as Danielle and Mary, reported a decline in enjoyment, explaining how the MERP initially made their meals less enjoyable. Danielle felt that the focus on the mindful eating questions took away from the focus on the food as a pleasure, she suggests that it is the task of reading that reduces enjoyment and that taste is still a primary aspect of enjoyment in food. For Mary, the focus on food was unhelpful because the food lacked taste and texture and the mindful eating questions drew more attention to the lack of sensory elements of the food.

*Danielle [33 years] (CO): “Nine times out of ten you're eating food that you like and that you like the taste of, but if I’m having to sit there and read something it's taking away from that enjoyment, so it's no longer an enjoyment of a process, it was a chore - (76.17)”*

*Mary [45 years] (CO): “It made it less enjoyable to begin with because it's like a lot of the foods are bland (.) in colour and it all looks the same - (98.21)”*

Whilst there were differing accounts of whether and how using the MERP impacted on enjoyment, there was a shared understanding that taste and texture were primary, important elements of the food experience.

### Master theme: MERP usage and development

The final theme further provides insight into participants’ experiences with the MERP, highlighting some barriers to utilising the tool. Danielle, Claire, and Mandy express concerns about using the MERP during social meals, as it may disrupt the usual sociability and conversation that accompany such occasions. This emphasizes that eating is a social activity and the importance of considering this more fully using mindfulness tools.

*Danielle [33 years] (CO): “I’ve got like children and they’re always on at me; it doesn’t fit a family atmosphere, I don’t think - (10.2)”*

*Claire [38 years] (CO): “I think during our mealtimes, they're always quite sociable, so having that [the MERP], and concentrating on that took away from that [the social aspect of eating], and that was a bit of a disadvantage for me - (24.6.7)”*

*Mandy [50 years] (CO): “I could find it helpful, but at the same time, sometimes eating alone is not a nice thing to do; you can get lonely or sometimes you need to talk to somebody - (66.15)”*

The disadvantages of using the MERP while eating socially also impact usage

patterns. While there were differences in when and how participants used the MERP, the primary considerations were often about who they were eating with, and where they were eating, with a preference for using it at home rather than at work. This may be indicative of the embarrassment of using tools to regulate eating in front of other people which aligns with feelings of weight shame. The aspect of enjoyment was also integral in usage, where mealtimes that were more relaxed and focused were preferred for using the MERP

*Lynn [47 years] (CO): “Just breakfast and lunch mainly [that Lynn used the MERP], but if we had family over for dinner, then it was just not an option - (34.6–7)”*

*Hannah [31 years] (PBS): “Because my breakfast is just a bog-standard yoghurt and a hot drink so not really much thinking to be done for my breakfast – (32.10)”*

*Jenny [52 years] (CO): “I was eating, but because lunchtime I was working, so it's a desk meal, I wasn’t using the card. I was only using the card at home in the evening meal - (4.1)”*

*Debbie [48 years] (CO): “I tried to use it more of an evening meal because that's when I usually feel most relaxed. In the evening, it's almost that I can sit and enjoy my food - (38.50)”*

This barrier to using the MERP socially was reduced once the need for a physical copy of the instructions was perceived to be less necessary. The MERP was used less frequently as participants became more accustomed to mindful eating practices and internalized the principles. This decrease in usage was seen as a natural progression and may have facilitated using mindful eating in more diverse spaces although for some, the lack of engagement with a prompt did result in less use.

*Kerry [60 years] (CO): “At the start, I was using it, you know, every meal. Then after a few days when I’d got into the routine of being mindful and doing whatever, then I didn’t need to have it in front of me - (30.5)”*

*Katie [63 years] (PBS): “Because they became a bit familiar and I thought I don’t need it [the MERP] I know what I am doing and then without having it there in front of you you begin to slip a little bit – (46.17)”*

To overcome these barriers and to further aid the usability of the MERP, some participants suggested the potential benefits of an electronic format or app, allowing for discreet and convenient access during meals. They proposed features like audio prompts and interactive elements to enhance the user experience.

*Mary [45 years] (CO): “That would be easier [electronic format / app] because you’d just have it [mobile phone] on the table, and only you would know, and it's something you can keep looking at because you always have your phones on you - (140.31)”*

*Becky [61 years] (CO): “Do you know like just instead of having a card, actually hearing somebody, something to say - (29.6)”*

Overall, the analysis provides valuable insights into the challenges and benefits of using the MERP for mindful eating and offers suggestions for its improvement, including addressing issues related to usability and social dynamics during meals.

Before concluding the results section, it is important to note the similarities and differences between clinical obesity (CO) and post-bariatric surgery (PBS) patients regarding their experiences with the Mindful Eating Reflection Practice (MERP). Both groups reported heightened awareness and self-regulation due to the MERP, which led to healthier and more adaptive eating habits and choices. However, there were notable differences in their specific experiences with mindful eating. CO participants primarily focused on internal cues and portion control, whereas PBS participants placed more emphasis on appreciating the sensory elements and overall enjoyment of food. Despite these differences, both groups encountered similar challenges when using the MERP in social settings. Participants suggested improvements that reflected a need for more discreet and user-friendly formats, such as electronic versions of the MERP.

## Discussion

The participant quotes and the IPA presented in this study provide valuable insights into the profound impact of mindful eating and self-regulation on individuals’ eating behaviours. This investigation has unveiled a set of salient themes that contribute to a nuanced understanding of the role of the Mindful Eating Reflective Practice (MERP) in shaping eating practices.

One prominent theme that emerged from the analysis is the heightened awareness of internal cues. Participants’ experiences, as expressed in their statements, demonstrate that the MERP has prompted them to become more attuned to their body's signals of hunger and fullness. This heightened internal awareness has translated into more informed and mindful eating decisions, aligning their consumption patterns with their physiological needs (Elliston et al., 2017; Willem et al., 2019). The findings underscore the notion that mindful eating plays a pivotal role in fostering individuals’ trust in their bodies and guiding them towards more adaptive eating behaviours, but importantly, by changing the eating behaviour predicting an enhanced decision-making capability around food to allow for eating mindfully. There is a need for researchers and clinicians to consider a shift in how mindful eating is viewed in reflecting on the current findings. Mantzios (2021) suggests using the term “mindful eating behavior” as it better reflects the scientific nature of mindfulness practices. This is relevant to the present research and the use of the MEBP as a mindful eating behavior intervention, as even though it does not focus on internal cues such as hunger and satiety, and participants do not receive specific psychoeducation on them, they are still enhanced. This shift highlights that mindful eating might solely be about the mindful relationship with food that influences awareness of internal cues and corresponding decision-making, rather than awareness of internal cues having a primary stage in mindful eating programmes. In other words, while internal cues are important, focusing on observable mindful eating behaviors is the beginning and an indirect method of enhancing internal cues, which might be a more practical way to design and evaluate interventions, and a more sustainable method for people to engage with the interventions.

Secondly, this study illuminates the MERP's role in fostering healthier and more adaptive eating practices. Participants’ narratives, as reflected in their testimonies, revealed a shift away from less nutritious dietary choices towards fresh, wholesome options. The MERP appeared to act as a catalyst for mindful decision-making, nudging individuals towards healthier consumption patterns (Czepczor-Bernat et al., 2020; Keirns et al., 2022). In the context of behavioural nudging, the mindful eating intervention employed in this study effectively nudged participants towards adopting more mindful eating behaviour. This shift towards mindful eating, driven by the intervention's gentle nudging, underscores the potential of such interventions to facilitate positive behavioural changes and promote healthier eating practices. Additionally, the theme of slowing down consumption suggests that mindful eating interventions encourage individuals to adopt a more measured pace when eating, fostering a mindful and deliberate approach to their meals. Such outcomes are worth exploring on a larger scale in future research, and the potential of outcomes that are significant in health behaviour change.

A third noteworthy theme centres on the sensory attention to food. Several participants in the study articulated a transformation in their relationship with food, emphasizing their increased appreciation for the sensory qualities of their meals (Schnepper et al., 2019). The taste, texture, and aroma of food became pivotal points, resulting in an enhanced overall enjoyment of eating. This finding underscores the potential of mindfulness interventions, such as the MERP, to elevate the act of eating from a utilitarian necessity to a source of sensory delight. It highlights how mindful eating encourages individuals to savour their meals, making eating a more mindful and pleasurable experience (Stevenson et al., 2015). Importantly, such findings illustrate the potential for enhancing sensory-specific satiety is not only more aligned with mindfulness and mindful eating theory and practice (Bishop et al. 2004; Kabat-Zinn, 2003; Mantzios, 2023; Shapiro et al., 2006), but also provides a new direction in mindful eating research and interventions.

When individuals consume food, their liking for a specific food tends to decrease, a phenomenon termed sensory-specific satiety (Rolls et al., 1981). This concept serves as a critical factor in determining the cessation of eating (Hetherington, 1996). An important question arises as to whether individuals vary in how they employ their pleasurable feelings regarding food, with and without mindful eating, and in comparison, to internal cues of physical hunger and fullness. Research conducted by Hetherington (1996) and Mook and Votaw (1992) suggests that some people rely on the enjoyment they derive from food, while others depend on physiological signals from their bodies to decide when to stop eating. The reasons individuals determine when to conclude their meals or snaking (Chawner et al., 2022; Cunningham et al., 2021) have significant implications for mindful eating practices and suggest a method of further advising theories on mindful eating, and clinical and non-clinical interventions. Moreover, a cross-cultural study (Hyldelund et al., 2022) examined food consumer behaviour in samples from China and Denmark. It identified “Sensory-driven Pleasure” as the primary driver of food pleasure in both cultures, suggesting a universal emphasis on sensory aspects (Hyldelund et al., 2021). The interlink between enhancing sensory experiences through mindful eating, the implications for determining cessation of eating, and the associated pleasure of food may be one of the most exciting directions of eating in moderation, and will provide essential knowledge for eating researchers, clinicians and industry alike.

### Limitations

A criticism of the study was the female-centric sample, limiting generalisability, but consistent with clinic numbers that are 70–80% women. Although IPA encourages a fairly homogenous sample, data derived from male participants could have generated different insights. However, the under-recruitment of male participants is commonplace, with studies suggesting that men are underrepresented in weight loss (Robertson et al., 2016) and mindfulness research (Chin et al., 2019). To mitigate these issues and encourage male participation, research has suggested that interventions should be designed and tailored to specifically attract, engage and retain men, as few trials have consulted men during the development of interventions (Robertson et al., 2016). Also, the voluntary participation led to a non-representative sample of Heartlands Hospital, which serves a multi-ethnic and diverse population, and findings are restricted to white participants. Socio-ecoomic status, gender and racial or ethnic differences, preferences for foods and applicability of interventions largely depend on diverse populations (Jang and Vorderstrasse, 2019; Rao and Donaldson, 2015; Robertson et al., 2016), with arguments leaning towards a more mandatory participation model in health services (Cheung et al., 2017). Additional consideration of the findings should be given to the relatively small sample size, with future directions potentially focusing on multiple treatment hospitals with an aim of enhancing the generalizability and robustness of present research findings.

Another methodological consideration of the present study is the co-occurrence of the UHB NHS Trust programme. All participants who chose to take part in the interviews were enrolled on the hospital programme, which may have confounded/influenced accounts given during the interview. Although participants were advised that the purpose of the interview was to explore their experience of using the MERP, naturally there may have been a cross-fertilisation of practices and effects considering that both the MERP and the UHB NHS Trust programme related to eating and weight regulation. But there were several strengths as well. First, the study provides a contextual understanding of the broader environment in which the study is situated. This context can be essential for interpreting findings and considering the broader implications of the MERP within the healthcare system. Second, the study design allows for a potential comparison between the experiences of individuals using the MERP and those not engaged in the UHB NHS Trust program. This comparative analysis could reveal nuanced differences in perceptions, challenges, and successes, providing a more comprehensive view of the factors influencing eating and weight regulation. Third, individuals enrolled in a hospital program may enhance the generalizability of findings to similar populations or settings. This can be particularly valuable for informing interventions and policies in healthcare contexts addressing eating and weight regulation.

### Clinical implications

The findings offer promise for clinicians seeking to integrate mindful eating practices into their interventions for weight management and overall well-being. This study highlights the potential of Mindful Eating Reflective Practice (MERP) as a valuable tool for clinicians working with individuals seeking to improve their eating behaviors. The findings suggest that MERP can be effective in (a) Enhancing sensory appreciation of food, which is described as a heightened appreciation for the sensory qualities of food, potentially increasing enjoyment and promoting satiety; (b) Heightening awareness of internal cues, where participants reported becoming more attuned to hunger and fullness signals, leading to more informed eating decisions; (c) Promoting healthier eating patterns seen through a shift towards more nutritious choices and mindful decision-making around food consumption; (d) Encouraging mindful eating, where the intervention appeared to nudge participants towards slowing down and savoring their meals, fostering a more deliberate approach to eating. A large clinical randomised trial, overcoming limitations of gender bias and confounding elements such as receiving additional interventions that are part of the standard treatment would shed more light on the clinical utility of the MEBP.

In conclusion, this analysis underscores the transformative potential of mindfulness and self-regulation in reshaping individuals’ eating behaviours. The MERP, as an embodiment of mindful eating in a practical tool, demonstrates its efficacy in promoting internal awareness, an enhanced eating experience, and as an outcome, healthier dietary choices. These findings contribute to the growing body of literature on the role of mindfulness and mindful eating in promoting healthier, more mindful, and more satisfying eating practices, with implications for both clinical and public health interventions.
